# Stable States of a Microbial Community Are Formed by Dynamic Metabolic Networks with Members Functioning to Achieve Both Robustness and Plasticity

**DOI:** 10.1264/jsme2.ME23091

**Published:** 2024-03-28

**Authors:** Masahiro Honjo, Kenshi Suzuki, Junya Katai, Yosuke Tashiro, Tomo Aoyagi, Tomoyuki Hori, Takashi Okada, Yasuhisa Saito, Hiroyuki Futamata

**Affiliations:** 1 Graduate School of Science and Technology, Shizuoka University, Hamamatsu, Hamamatsu 432–8011, Japan; 2 Microbial Ecotechnology, Department of Biotechnology, Graduate School of Agricultural and Life Sciences, The University of Tokyo, 111 Yayoi, Bunkyo-ku, Tokyo, Japan; 3 Department of Applied Chemistry and Biochemical Engineering, Graduate School of Engineering, Shizuoka University, Hamamatsu, 432–8011, Japan; 4 Environmental Management Research Institute, National Institute of Advanced Industrial Science and Technology (AIST), 16–1 Onogawa, Tsukuba, Ibaraki 305–8569, Japan; 5 Institute for Life and Medical Sciences, Kyoto University, Kyoto, 606–8507, Japan; 6 Department of Mathematics, Shimane University, Matsue, 690–8504, Japan; 7 Research Institution of Green Science and Technology, Shizuoka University, Shizuoka 422–8529, Japan

**Keywords:** stability, metabolic network, robustness, plasticity

## Abstract

A more detailed understanding of the mechanisms underlying the formation of microbial communities is essential for the efficient management of microbial ecosystems. The stable states of microbial communities are commonly perceived as static and, thus, have not been extensively examined. The present study investigated stabilizing mechanisms, minority functions, and the reliability of quantitative ana­lyses, emphasizing a metabolic network perspective. A bacterial community, formed by batch transferred cultures supplied with phenol as the sole carbon and energy source and paddy soil as the inoculum, was analyzed using a principal coordinate ana­lysis (PCoA), mathematical models, and quantitative parameters defined as growth activity, community-changing activity, community-forming activity, vulnerable force, and resilience force depending on changes in the abundance of operational taxonomic units (OTUs) using 16S rRNA gene amplicon sequences. PCoA showed succession states until the 3^rd^ transferred cultures and stable states from the 5^th^ to 10^th^ transferred cultures. Quantitative parameters indicated that the bacterial community was dynamic irrespective of the succession and stable states. Three activities fluctuated under stable states. Vulnerable and resilience forces were detected under the succession and stable states, respectively. Mathematical models indicated the construction of metabolic networks, suggesting the stabilizing mechanism of the community structure. Thirteen OTUs coexisted during stable states, and were recognized as core OTUs consisting of majorities, middle-class, and minorities. The abundance of the middle-class changed, whereas that of the others did not, which indicated that core OTUs maintained metabolic networks. Some extremely low abundance OTUs were consistently exchanged, suggesting a role for scavengers. These results indicate that stable states were formed by dynamic metabolic networks with members functioning to achieve robustness and plasticity.

Changes in and the stability of microbial communities are well-known phenomena and some of the most important features in complex, engineered, and synthetic microbial communities (SMCs). These features are observed as community succession and stable states, *e.g.*, a planktonic bacterial community exhibited these states repeatedly in batch and chemostat cultures (Futamata *et al.*, 2005; Aziz *et al.*, 2015; Suzuki *et al.*, 2018), which are considered to be dependent on microbial adaptive processes to the changing environments of outer/inner cells and interspecies interactions (Sanchez-Gorostiaga *et al.*, 2019; Ortiz *et al.*, 2021; Daniels *et al.*, 2022). Microbial dynamics have been extensively examined under culture-dependent (Friedman *et al.*, 2017) and -independent (Reiss *et al.*, 2016) as well as mathematical conditions (Smith, 2011); however, the mechanisms underlying community succession and stable states remain unclear. Community dynamics are not necessarily explained by kinetic and growth parameters that are conventionally regarded as the factors controlling complex microbial communities, such as bioreactors (Futamata *et al.*, 2005; Aziz *et al.*, 2015). Clarifying the microbial dynamics is experimentally and mathematically in progress. Therefore, the practical application of microbes to the management of microbial communities, characterized by community succession and stable states, remains a challenging subject that warrants further study on the underlying mechanisms.

The processes by which a microbial community forms are important for the suitable management of microbial ecosystems, *i.e.*, wastewater treatment, agriculture fields, and the gut microbiota (Luo *et al.*, 2022). The high complexities of microbial ecosystems are too high to analyze. SMCs offer a valuable approach to investigating bacterial population dynamics by reducing complexity and increasing controllability (Haruta *et al.*, 2002, 2018; Kato *et al.*, 2005; Narisawa *et al.*, 2008; De Roy *et al.*, 2014; Mee *et al.*, 2014; Aziz *et al.*, 2015, 2021; Friedman *et al.*, 2017); however, the simplicity of SMCs may lead to crucial factors being overlooked (Altmeyer and McCaskill, 2001), such as the effects of minor populations and microbial diversity on the formation of microbial communities. It is challenging to examine the effects of minor populations on the dynamics of whole systems because isolated bacterial strains corresponding to a minor population in a complex system may not necessarily become a minority in a SMC. This is a fundamental problem of reductionist approaches. As another approach, community networks have been applied to examine the features of microbial complex systems, with a focus on the properties of network topologies without interspecies interactions (Cao *et al.*, 2018; Ritter *et al.*, 2021; Guo *et al.*, 2022); however, the mechanisms underlying community dynamics have yet to be discussed in detail. Novel approaches are need to‍ ‍obtain insights into microbial complex systems by combining the dynamics of a whole system and a distinct population with quantitative evaluations.

The present study investigated how microbial complex systems maintain a stable community structure in order to obtained a more detailed understanding of stabilizing mechanisms for not only industrial producing processes, but also managing microbial ecosystems. However, the stable states of microbial communities are considered to be static and, thus, have not been extensively examined. To resolve the fundamental problem described above, we herein analyzed a bacterial community in transferred cultures by combining 16S rRNA gene amplicon sequence ana­lyses with mathematical simulation and quantitative ana­lyses. Many types of bacteria experimentally and theoretically coexist even with limited resources and competition (Futamata *et al.*, 2001b, 2005; Smith, 2011; Wang and Liu, 2020), conditions under which the metabolic network may play an important role in bacterial coexistence because metabolites support cell growth (Daniels *et al.*, 2022). The metabolic network is important for understanding formation mechanism and coexistence of bacterial community (Aziz *et al.*, 2015; D’Souza *et al.*, 2018; Goldford *et al.*, 2018; Alnahhas *et al.*, 2020). From the viewpoint of metabolic networks, we herein discussed stabilizing mechanisms under stable states of the microbial community, the functions of minorities, and the validity of quantitative ana­lyses.

## Materials and Methods

### Transferred batch cultures

MP medium (Watanabe *et al.*, 1998) containing (L^–1^) 2.75‍ ‍g of K_2_HPO_4_, 2.25‍ ‍g of KH_2_PO_4_, 1.0‍ ‍g of (NH_4_)_2_SO_4_, 0.2‍ ‍g of MgCl_2_ 6H_2_O, 0.1‍ ‍g of NaCl, 0.02‍ ‍g of FeCl_3_ 6H_2_O, and 0.01‍ ‍g of CaCl_2_ and supplemented with 0.2‍ ‍g of phenol (pH 6.8 to 7.0) was used for transferred batch cultures. An elementary flask (volume of 100‍ ‍mL) containing 18‍ ‍mL of MP medium was autoclaved at 121°C for 15‍ ‍min, and 2‍ ‍mL of a paddy soil suspension (20‍ ‍g soil in 20‍ ‍mL of MP medium) was then added to the flask. Phenol was sterilized by filtration (membrane filters with a pore size of 0.2‍ ‍μm; ADVANTEC) and then added at 0.2‍ ‍mM as the sole carbon and energy source. The culture was incubated at 25°C with rotary shaking at 125‍ ‍rpm. Phenol in the culture was measured using a colorimetric assay with a Phenol Test Wako kit (Fujifilm Wako Pure Chemical) (Futamata *et al.*, 2001b), the detection limit of which was approximately 1.0‍ ‍μM. When phenol was not detected, 2‍ ‍mL of a parent culture was transferred into a flask containing 18‍ ‍mL of fresh MP medium. Phenol was not detected after a 24-h incubation in all cultures. The batch culture was enriched up until the 15^th^ transfer. All cultures were conducted in triplicate.

### Amplification and purification of 16S rRNA genes

DNAs extracted from transferred batch cultures were used in the present study, and the V4 region of the 16S rRNA gene was amplified with DNA polymerase Q5 (New England BioLabs) and the universal primers sets of *515F* (5′-GTGCCAGCMGCCGCGGTAA-3′) and *806R* (5′-GGACTACHVGGGTWTCT AAT-3′). The primer of *515F* had the adaptor region of the Illumina P5 sequence, while *806R* had the adaptor region of the Illumina P7 sequence with 12 bp barcodes (Caporaso *et al.*, 2012). PCR was conducted under the following conditions: preheating at 98°C for 90‍ ‍s, followed by 25 cycles of denaturation at 98°C for 10‍ ‍s, annealing at 58°C for 30‍ ‍s, and extension at 72°C for 30‍ ‍s in each cycle, and a final extension at 72°C for 2‍ ‍min. Amplicons were purified with the AMPure XP kit (Beckman Coulter) according to the manufacturer’s instructions. The purified DNA solution was electrophoresed, and the DNA fragment (approximately 250 bp) was cut from the gel and purified with the Wizard SV Gel and PCR Clean-Up system (Promega) according to the manufacturer’s instructions. The concentration of purified DNA was measured with the Quant-iT PicoGreen ds DNA reagent and kit (ThermoFisher Scientific).

### Illumina sequencing and data processing

The barcode-encoded DNA library and initial control (PhiX; Illumina) were subjected to paired-end sequencing with a 300-cycle MiSeq Reagent kit (Illumina) on a MiSeq sequencer (Illumina). PhiX, low-quality (Q<30), and chimeric sequences were removed, and paired-end sequences were assembled as previously described (Itoh *et al.*, 2014). The sequences in each library were characterized phylogenetically using QIME software package version 1.7.0 (Caporaso *et al.*, 2010). Operational taxonomic units (OTUs) were grouped using a 97% sequence identity cut-off. Alpha-diversity indices (Chao1, Shannon, and Simpson reciprocal) and beta-diversity indices (weighted UniFrac distances for a principal coordinate ana­lysis [PcoA]) were calculated using the QIME program (Caporaso *et al.*, 2010; Lozupone *et al.*, 2011). Representative sequences for each OTU were assigned using BLAST in the DDBJ nucleotide sequence database. Bacteria with a relative abundance of <0.01% to total bacteria were grouped into the same cluster.

### PCoA ana­lysis of the bacterial community structure

PCoA was performed based on the Bray-Curtis index with the abundance of OTU because this index has been recognized as one of the most useful methods for evaluating differences among populations (Faith *et al.*, 1987; Clarke, 1993). The following equation was used to calculate the Bray-Curits index.

$$\delta_{AB}=\sum \left|n_{A}-n_{B}\right|∕\sum \left(N_{A}+N_{B}\right)\;0\;\leq \delta_{AB}\leq 1$$

where *δ_AB_* is the dissimilarity index between the communities of A and B, *n_A_* and *n_B_* is the abundance of OTUs in samples of A and B, respectively, and *N_A_* and *N_B_* is the total abundance of OTUs in samples of A and B, respectively. The weighted UniFrac distance was calculated using the abundance of OTUs as described above.

### Activities and vulnerable and resilience forces

We developed equations to quantitatively evaluate bacterial dynamics. The abundance of an OTU indicates the relative population density of the bacterium corresponding to the OTU. A change in abundance indicates the relative growth activity of the OTU. The growth activity of an OTU was introduced and was defined as:

Growth activity=Xi(t+1)/Xi(t)

 (equation 1),

in which *t* is t^th^ transferred cultures, and Xi(t) and Xi(t+1) are the relative abundance of OTU_Xi in “t^th^” and “(t+1)^th^” transferred cultures, respectively.

To quantify the population-level contribution of growth activity, the population growth activity of OTU_Xi (PGA) in the (t+1)^th^ transferred culture was introduced and defined as:

$$\text{PGAi}=\{Xi(t+1)/Xi(t)\}\times Xi\left(t+1\right)$$

 (equation 2).

Since a microbial community is formed by populations, the community-forming activity of “(t+1)^th^” transferred culture was introduced and defined as the total sum of PGAi in the “(t+1)^th^” transferred culture:

$$\text{Community-forming activity}=\sum_{i=1}^{n}PGAi$$

 (equation 3).

Community-changing activity was introduced and defined as equation (4), which was modified from the equation described by Fisher and Mehta (Fisher and Mehta, 2014) as follows:

$$\text{Community-changing activity}=\sum_{i=1}^{n}{\left[{loɡ}_{10}\{Xi(t+1)/Xi(t)\}\right]}^{2}$$

  (equation 4).

To investigate ecological stability, a metric termed “specific vulnerable/resilience force” was developed and defined as:

$$\left\{{Loɡ}_{10}\frac{Xi\left(t+2\right)}{Xi\left(t+1\right)}-{Loɡ}_{10}\frac{Xi\left(t+1\right)}{Xi\left(t\right)}\right\}/\{{Loɡ}_{10}Xi\left(t+1\right)-{Loɡ}_{10}Xi\left(t\right)\}={Loɡ}_{10}\frac{Xi(t+2)\times Xi(t)}{{\left\{Xi\left(t+1\right)\right\}}^{2}}/{Loɡ}_{10}\frac{Xi(t+1)}{Xi(t)}\;$$

 (equation 5),

in which positive and negative values indicate vulnerable and resilience forces, respectively. A more positive value means a stronger vulnerable force, while a more negative value means a stronger resilience force (Supplementary Fig. S1). The population-level and community-level contributions of the specific vulnerable/resilience forces were introduced and defined as equations 6 and 7, respectively.

$$\left\{{Loɡ}_{10}\frac{Xi(t+2)\times Xi(t)}{{\left\{Xi\left(t+1\right)\right\}}^{2}}/{Loɡ}_{10}\frac{Xi(t+1)}{Xi(t)}\right\}\times \left\{\frac{Xi\left(t\right)+Xi\left(t+1\right)+Xi(t+2)}{3}\right\}$$

 (equation 6)

$$\sum_{i=1}^{n}\left[\left\{{Loɡ}_{10}\frac{Xi(t+2)\times Xi(t)}{{\left\{Xi\left(t+1\right)\right\}}^{2}}\;/\;{Loɡ}_{10}\frac{Xi\left(t+1\right)}{Xi\left(t\right)}\right\}\times \left\{\frac{Xi\left(t\right)+Xi\left(t+1\right)+Xi\left(t+2\right)}{3}\right\}\;\right]$$

 (equation 7)

### Simulation

Common OTUs were selected in one of the three replications at least from the soil sample to the 15^th^ transferred culture, and the growth activities of common OTUs were analyzed to characterize the dynamics of OTUs. According to the features of OTU dynamics (Supplementary Fig. S2), we conducted a numerical simulation of the consumer-resource model with resource leakage, which accounted for metabolic network effects (Marsland *et al.*, 2019). It monitored the concentrations of phenol and catechol in cultures, but generally not other metabolites. The types of phenol and catechol utilizers may be enriched, while those of other metabolite utilizers may be more diverse because various metabolites were present in transferred batch cultures supplied with phenol as the sole carbon source. Therefore, species were segmented into three distinct groups for the incorporation of metabolic leakage: phenol-utilizing specialists (50 species), secondary product-utilizing specialists (50 species), and other species (100 species). The model is given by

$$\frac{dN_{i}}{dt}={ɡ}_{i}\;N_{i}\;\left(J_{i}^{grow}-m_{i}\right)$$,

$$\frac{dR_{a}}{dt}=\kappa_{a}-\frac{R_{a}}{\tau_{R}}+\sum_{j=1}^{S}N_{j}\left(J_{ja}^{out}-J_{ja}^{in}\right)/w_{a}$$

where *ɡ_i_* is a proportionality constant of the growth rate, *m_i_* is a parameter corresponding to the minimum energy requirement. $J_{i}^{grow}$, $J_{ja}^{out}$, and $J_{ja}^{in}$ represent the energy flux for growth, the outgoing flux, and the incoming energy flux, respectively. Specifically, $J_{ja}^{in}$ is defined as

$$J_{ja}^{in}=w_{a}c_{ia}R_{a}$$

where *w_a_* denotes the energy density of resource *a*, and *c_ia_* (for fixed *i*) represents a vector of the consumer’s preference for resources. A fraction *l_a_* of the imported flux $J_{ia}^{in}$ is secreted into the environment in the form of resource *b*, while the remainder is used for growth:

$$J_{ib}^{out}=\sum_{a=1}^{R}{D_{ba}l}_{a}J_{ia}^{in}$$

$$J_{i}^{grow}=\sum_{a=1}^{R}(1-l_{a})J_{ia}^{in}$$

Here, *D_ba_* is a matrix representing the fraction of energy flux from resource *a* secreted in the form of resource *b* ($\sum_{b=1}^{R}D_{ba}=1$) (Supplementary material S1). The classic consumer-resource model (MacArthur, 1970), in which metabolic cross-feeding is not accounted for, is obtained by setting *l_a_*=0. To replicate our batch-culture experiment settings, we set the external supply to zero (κ*_a_*=0) and omitted the dilution term $R_{a}/\tau_{R}$. Given the unavailability of resource abundance data from our experiment, we adopted a parsimonious assumption that the number of resources, *R*, was two, *i.e.*, phenol and its metabolites. To implement a batch-culture scenario, 10% of *N_i_* and *R_a_* were taken at the end of each cycle and transferred to the next cycle along with a specified amount of a single resource, phenol. We recorded the abundance of each species at the end of each cycle. A rank-abundance distribution was simulated using the consumer-resource model with resource leakage (Marsland *et al.*, 2019).

### Accession number

The nucleotide sequences of the 13 core OTUs (described below) reported in the present study have been deposited in the GSDB, DDBJ, EMBL, and NCBI nucleotide sequence databases under accession numbers LC785368 to LC785380.

## Results

### Bacterial community dynamics and community structure

**Fig. 1.**
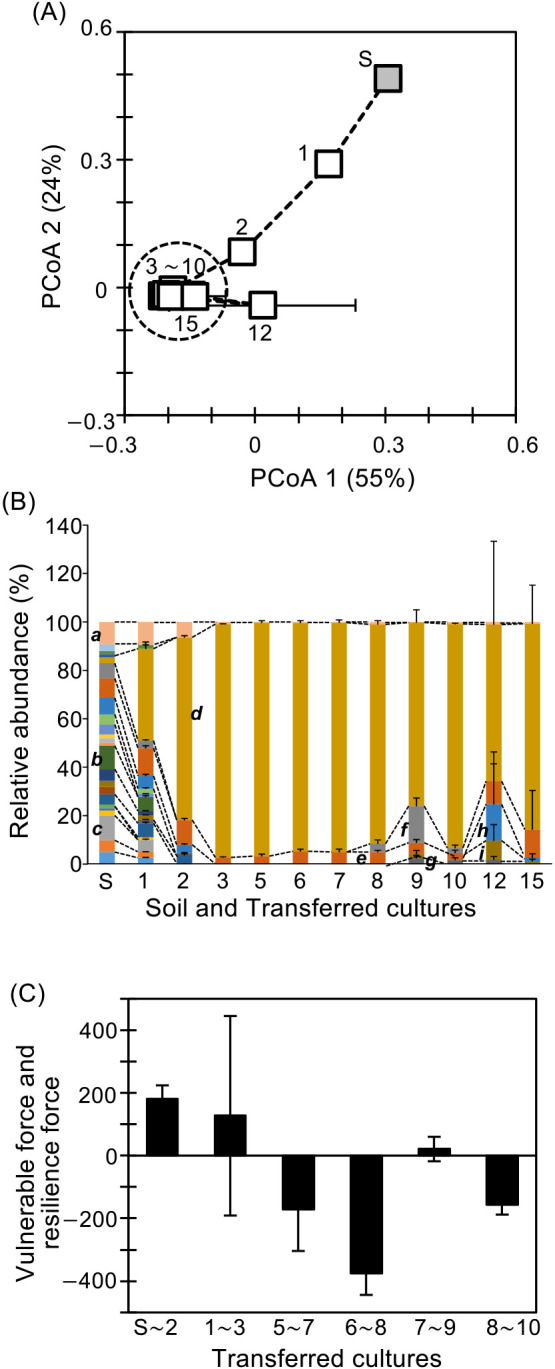
Dynamics of a bacterial community. (A) PCoA with weighted UniFrac distances. Gray square: soil used as the inoculum, open square: batch transferred cultures; the dotted circular line indicates the bacterial community under stable conditions. “S” and the number beside the open square indicate the soil sample and order of transferred cultures, respectively; (B) Community structure of phylum and *proteobacteria* levels. *a*: bacteria with less than 1% abundance, *b*: *Anaerolineae*, c: *Acidobacteria*, d: *γ-proteobacteria*, *e*: *β-proteobacteria*, *f*: *δ-proteobacteria* *g*: *Flavobacteriia*. *h*: *α-proteobacteria*, *i*: *Sphingobacteriia*. Error bars represent standard deviations (*n*=3); (C) The vulnerable force and resilience force of a community enriched in transferred cultures of Soil~2^nd^, 1^st^~3^rd^, 5^th^~7^th^, 6^th^~8^th^, 7^th^~9^th^, and 8^th^~10^th^. Positive and negative values indicate vulnerable force and resilience force, respectively (Supplementary Fig. S1). A more positive value indicates a stronger vulnerable force, while a more negative value indicates a stronger resilience force.

Batch transferred cultures were constructed with paddy soil as the inoculum and phenol as the sole carbon and energy source. Every 24 h, 10% of the parent culture was transferred into a fresh culture, which was conducted until the 15^th^ transferred culture with 3 replications. The dynamics of the bacterial community were analyzed with weighted UniFrac distances (Fig. 1A). PCoA ana­lyses showed that bacterial community succession was observed until the 3^rd^ transferred culture, and was followed by stable states of the bacterial community from the 3^rd^ to 15^th^ transferred cultures, with the exception of the 12^th^ transferred culture (Fig. 1A). The weighted UniFrac distance was 0.040±0.069 among the bacterial community during the stable state from the 5^th^ to 10^th^ transferred cultures.

The results of 16S rRNA gene amplicon sequence ana­lyses revealed that the bacterial community of soil used as the inoculum mainly consisted of 24 phyla and *α*-, *β*-, *γ*-, and *δ-proteobacteria*, which occupied approximately 90.7% of the total read number. The remaining 9.3% of the total read number consisted of a bacterial group, which occupied less than 0.01% of total read number (Fig. 1B). The phyla and *proteobacteria* occupying more than 5% of the total read number were *Acidobacteria* (10%), *Anaerolineae* (9.7%), and *β*- (8.0%), *α*- (6.8%), and *δ-proteobacteria* (6.3%) in the inoculum. Bacterial community succession was observed until the 3^rd^ transferred culture, resulting in the dominance of *γ-proteobacteria*, which occupied 96±0.17% of the total read number in the 3^rd^ transferred culture, while *γ-proteobacteria* occupied approximately 2.5% of the total read number in the inoculum (Fig. 1B). *γ-proteobacteria* was dominant, occupying 93±4.0% of the total read number, with the exception of the 9^th^ and 12^th^ transferred cultures. Some phyla and *proteobacteria* showed partial occupation: *α-proteobacteria*, *δ-proteobacteria*, *Sphingobacteriia*, and *Flavobacteriia* occupied 8.1±9.7% from the 12^th^ to 15^th^, 6.8±7.2% from the 8^th^ to 10^th^, 3.6±3.8% from the 9^th^ to 15^th^, and 2.0±0.4% from the 9^th^ to 12^th^ transferred cultures, respectively (Fig. 1B), whereas *Flavobacteriia*, *Sphingobacteriia*, *Actinobacteria*, and *Bacilli* occupied 13±6.4% from the 5^th^ to 15^th^, 5.5±4.1% from the 5^th^ to 15^th^, 2.8±0.97% from the 7^th^ to 15^th^, with the exception of the 8^th^ and 9^th^, and 2.1±0.69% from the 6^th^ to 15^th^ transferred cultures, with the exception of 10^th^ and 12^th^, respectively (Fig. 1B). *β-proteobacteria* was constantly present during this experiment and occupied 6.7±3.3% of the total read number.

### Activity and forces of bacterial community dynamics

The results of PCoA showed bacterial community dynamics with community succession and stable states (Fig. 1A and B). Community-forming activity, community-changing activity, and vulnerable and resilience forces were evaluated to obtain a more detailed understanding of bacterial community dynamics (Table 1 and Fig. 1C). Common OTUs between (t) and (t+1) transferred cultures are needed to calculate these activities and forces as shown in equations (1–7). The percentage of common OTU abundance to total OTU abundance was >99% in all transferred cultures (Table 1), indicating that these activities and forces based on common OTUs mostly reflected total activities and forces. The average growth activity of all OTUs under succession states (from the 1^st^ to 3^rd^ transferred cultures) was lower than that of OTUs under stable states (from the 5^th^ to 10^th^ transferred cultures) (Table 1). Community-changing activity was higher under succession states than under stable states (Table 1). Community-forming activity under succession states was stable, but fluctuated under stable states (Table 1). The vulnerable and resilience forces of the community in transferred cultures were detected under succession and stable states, respectively (Fig. 1C). Quantitative data revealed the physiological conditions of bacterial community dynamics, which were not detected by PCoA ana­lyses.

### Simulation for the metabolic network

**Fig. 2.**
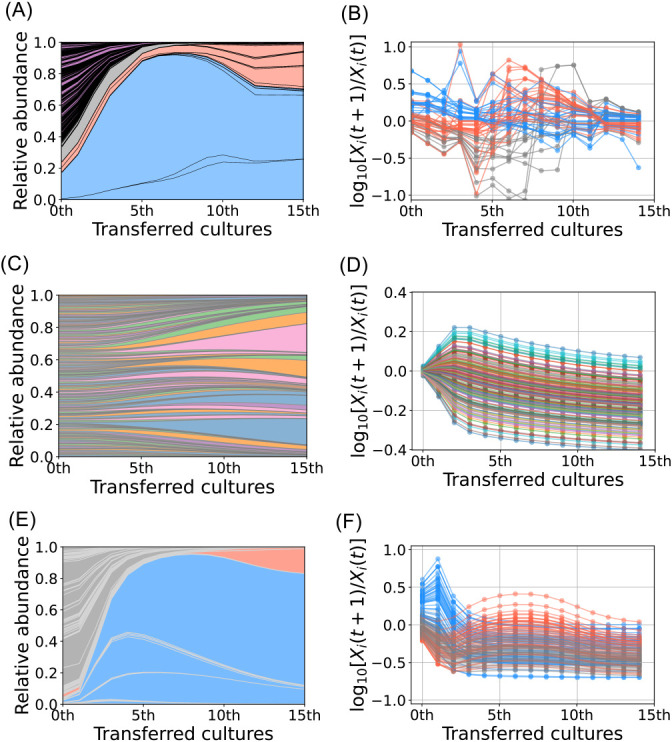
Abundance and growth activity of OTUs analyzed with experimental data and simulation models. (A) The time-series data of common OTU abundance based on these experimental data. Blue, red, and gray correspond to the colors shown in clustering ana­lysis data (Supplementary Fig. S2). Blue: Pronounced growth activity in the early stages, red: Pronounced growth activity in the later stages, gray: Negative growth activity throughout the experiment. Purple: Extinct OTUs. (B) Growth activity curve of common OTUs. Blue, red, and gray lines correspond to the colors shown in clustering ana­lysis data (Supplementary Fig. S2), which are the same as those shown in A. (C) The time-series data of OTU abundance simulated based on the classic consumer-resource model with a single resource (without leakage). (D) Growth activity curves computed from the simulated data shown in C. (E) Species abundance data obtained from the consumer-resource model with metabolic leakage. Bacterial species are classified into three groups: blue; phenol specialists (50 species), red; secondary-product specialists (50 species), and gray; other species (100 species). (F) Growth-activity curves computed from the simulated data in E.

A simulation was conducted to investigate whether a metabolic network occurred among the OTUs in transferred cultures. The abundance of common OTUs changed (Fig. 2A), and intersecting time-series curves of growth activities were observed using empirical data (Fig. 2B). The classic consumer-resource model (MacArthur, 1970) did not show significant changes in the relative abundance of OTUs (Fig. 2C) or the intersection of growth activity curves (Fig. 2D). These results indicate a feature of microbial dynamics that does not have a metabolic network. The intersections evident in our empirical data challenged this classic model, indicating the need to investigate other underlying mechanisms. We hypothesized that the metabolic network is a compelling mechanism to explain these observations. To validate this hypothesis, we simulated the consumer-resource model incorporating metabolic leakage (Marsland *et al.*, 2019) by segmenting species into three distinct groups: phenol-utilizing specialists (50 species), secondary product-utilizing specialists (50 species), and other species (100 species). The results obtained revealed that under specific parameter settings (Supplementary material S1), our simulation almost reproduced empirically observed changes in abundance and growth activity (Fig. 2E and F), suggesting that a metabolic network occurred among the OTUs in transferred cultures.

**Fig. 3.**
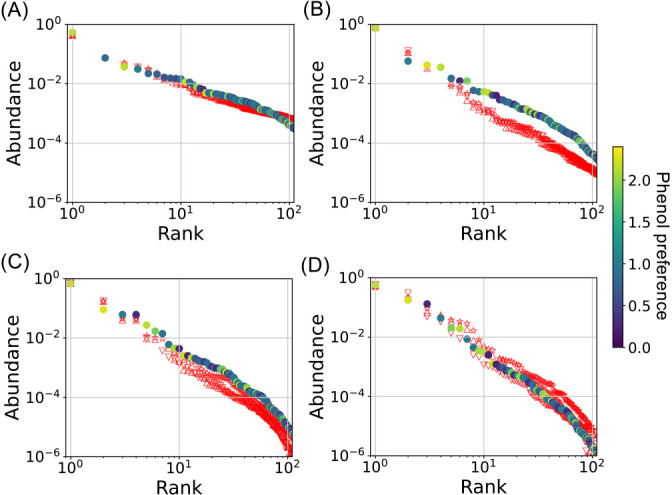
Rank-ordered frequency distribution of OTUs. The vertical axis indicates the relative abundance of OTUs. The horizontal axis indicates rank based on abundance. (A) The 2^nd^ transferred culture, (B) the 6^th^ transferred culture, (C) the 8^th^ transferred culture, and (D) the 10^th^ transferred culture. Simulated results are shown in filled circles, where the color indicates the phenol preference.

Additionally, we posited that the rank-abundance distribution may offer insights into metabolic features. To examine this, the rank-abundance distribution was computed using simulated abundance data in the 2^nd^, 6^th^, 8^th^, and 10^th^ cultures. The rank-abundance distribution obtained from the simulation aligned well with our experimental distributions (Fig. 3). The simulation results indicated that species with a higher preference for phenol were likely to dominate the higher ranks and *vice versa*, whereas all higher ranked species did not necessarily exhibit higher preference, with some showing lower preference in the 2^nd^, 6^th^, 8^th^, and 10^th^ cultures (Fig. 3).

### Dynamics of core OTUs and minorities

**Fig. 4.**
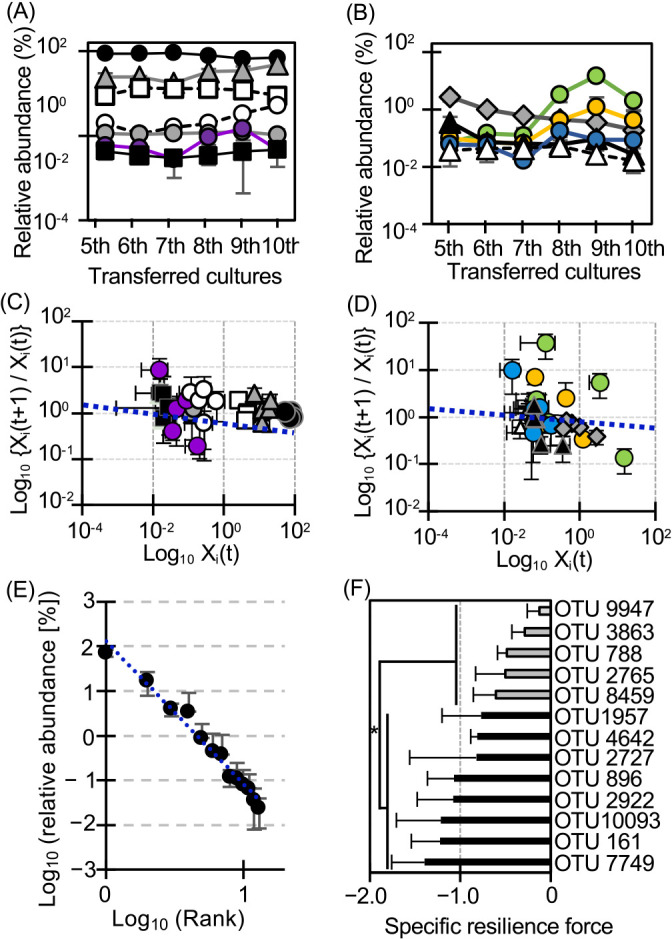
Population dynamics and resilience force of core OTUs under stable conditions from 5^th^ to 10^th^ transferred cultures. (A) and (B) Population dynamics of the 13 core OTU members. Closed black circle: OTU9947, gray triangle: OTU2765, open square: OTU2727, open circle: OTU1957, gray circle: OTU10093, purple circular: OTU2922, closed black square: OTU7749, Gray diamond: OTU3863, closed triangle; OTU896, green circle: OTU788, orange circle: OTU8459, blue circle: OTU161, open triangle: OTU4642. (C) and (D) Scatter plots of OTUs on Log_10_Xi(t) vs Log_10_{Xi(t+1)/Xi(t)}. Symbols indicate the same contents as those shown in (A) and (B). The gradient of the blue dotted line indicates the resilience force of the core member OTUs during stable conditions, which was –0.079. (E) Rank-abundance relationship of core OTUs. The average values of rank and abundance were used in a graph. The blue dotted line shows the power law with a correlation coefficient of 0.98. The gradient of the blue line was –3.2. (F) Specific resilience force of a distinct core member OTU during stable conditions. Force was calculated according to equation 5 described in the Materials and Methods section. *P*<0.05 (★).

To identify the stable conditions of a bacterial community, we focused on common OTUs that had coexisted during stable states from the 5^th^ to 10^th^ transferred cultures. Common OTUs consisted of 13 OTUs that were detected in all triplicate samples and occupied 98±1.4% of the total OTU abundance during stable states. This highlighted their roles as core OTUs, forming the primary framework of the bacterial community structure in stable states. Based on the distinct abundance of core OTUs, they were categorized into‍ ‍majorities (1%≤relative abundance), middle-class (0.1%≤relative abundance<1%), and minorities (0.01%≤relative abundance<0.1%) (Supplementary Table S1). An ana­lysis of core OTUs may provide insights into stable states because microbial coexistence is not natural, it occurs under specific conditions involving interspecies interactions, thereby affecting microbial metabolism and growth activity changes (Aziz *et al.*, 2015; Daniels *et al.*, 2022). The dynamics of distinct core OTUs differed from each other (Fig. 4A and B). OTU788 (the most closely related to *Bdellovibrio* sp.), OTU8459 (closely related to *Nubsella zeaxanthinifaciens*), and OTU1957 (*Pseudomonas putida*) increased, OTU896 (*Pseudomonas alcaligenes*) and OTU3863 (*Pseudomonas citronellolis*) decreased, while OTU161 (*Acinetobacter haemolyticus*) and OTU2922 (*Massilia guangdongensis*) fluctuated. The other OTUs were mostly maintained (Fig. 4A and B, and Supplementary Table S1). The rank-abundance distribution of the 13 core OTUs showed a power law trend (Fig. 4E). The average resilience force for the whole community was –0.079 under stable states (Fig. 4C and D), with each core OTU exhibiting specific resilience force (Fig. 4F). Force was grouped into two classes, with high and low activities at –‍1.0±0.23 and –0.40±0.19, respectively (Fig. 4F).

**Fig. 5.**
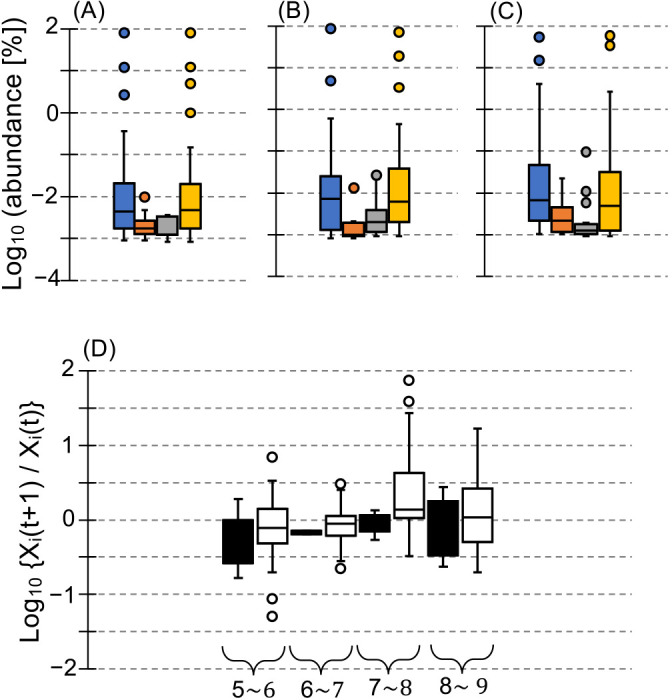
Dynamics and growth activity of bacterial populations. (A) From the 5^th^ to 6^th^ transferred cultures, (B) from the 7^th^ to 8^th^ transferred cultures, and (C) from the 9^th^ to 10^th^ transferred cultures. Blue bars indicate the abundance of total OTUs in parent cultures, red bars indicate abundance in parent cultures of OTUs not detected in transferred cultures, gray bars indicate the abundance of OTUs newly detected in transferred cultures, and yellow bars indicate the abundance of total OTUs in transferred cultures. (D) Comparison of the growth activity of disappeared OTUs with remaining OTUs. Black and white boxes indicate the growth activities of disappeared and remaining OTUs, respectively. The number at the bottom of the graph indicates the 5^th^~7^th^, 6^th^~8^th^, 7^th^~9^th^, and 8^th^~10^th^ transferred cultures, respectively. For example, the black box on the left side under the 5^th^~6^th^ transferred cultures indicates growth activities from the 5^th^ to 6^th^ transferred cultures of OTUs that survived in the 5^th^ and 6^th^ transferred cultures, but disappeared in the 7^th^ transferred culture, whereas the neighboring white box indicates growth activities from the 5^th^ to 6^th^ transferred cultures of OTUs that survived in the 5^th^, 6^th^, and 7^th^ transferred cultures, and likewise hereinafter.

Alternatively, population dynamics were analyzed at the OTU level (Fig. 5A, B, and C). The number of OTUs decreased and stabilized at 55±8.7 during stable states (5^th^ to 10^th^ transferred cultures) (Table 1 and Supplementary Fig. S4A). Approximately 32±7.5% of the OTUs present in parent cultures ([t] transferred cultures) were not detected in subsequent transferred cultures ([t+1] transferred cultures), whereas newly appeared OTUs occupied approximately 27±9.5% in newly transferred cultures ([t+1] transferred cultures) during stable states (Fig. 5A, B, and C, and Supplementary Fig. S4B and C). The combined abundance of undetected and newly detected OTUs was approximately 0.12% of the total abundance during stable states. The average abundances of these OTUs were approximately 0.0070 and 0.0083%, respectively (Fig. 5). The growth activities of undetected and remained OTUs were similar (Fig. 5D). These results suggest a consistent exchange involving extreme minorities, even during stable states.

## Discussion

A more detailed understanding of microbial community dynamics consisting of community succession and stable states is important for managing microbial ecosystems. The present study focused on stable states that have been recognized as static states without deep attention because they are valuable for productivity and functionality in natural and engineered microbial systems. In the present study, the dynamics of bacterial communities were evaluated using PCoA, mathematical models, and quantitative ana­lyses. The results obtained indicated that the stable states of bacterial communities were formed via dynamic metabolic networks with members functioning to achieve robustness and plasticity. This challenges our conventional understanding of microbial ecosystems.

Average growth activity, community-changing activity, and community-forming activity showed that bacterial communities are always in a dynamic state irrespective of succession or stable states recognized by PCoA. Vulnerable and resilience forces were useful parameters for quantitatively distinguishing which bacterial community was in a succession or stable state: bacterial communities were under stable states when resilience force was detected and *vice versa*. Quantitative ana­lyses showed that community-changing activity gradually decreased from approximately 180 to 27 during succession states, whereas community-forming activity was stable at approximately 180 with constant and low average growth activity at around 0.35. These results suggest that the majority of bacteria did not adapt well to new conditions (the carbon source was phenol), whereas specific bacterial groups adapted, grew constantly, and remained stable. In the succession state, *γ-proteobacteria* was dominant in the diverse soil bacterial community (Fig. 1B), providing quantitative insights into selection processes in new environments.

The three activities were not stable, but fluctuated under the stable states, which would be an important cue to understand them. Fluctuations in these activities suggest that the microbial community was formed via metabolic networks. Metabolic networks play roles in the functional stability of a whole system by supporting common goods (Morris *et al.*, 2013; Niehaus *et al.*, 2019; Smith and Schuster, 2019) and removing toxic metabolic byproducts that inhibit the growth of other bacteria (Luli and Strohl, 1990; Lilja and Johnson, 2016; Aziz *et al.*, 2021; Mohd Din *et al.*, 2021). Pure and synthetic microbial cultures collapse with feedback inhibition caused by the accumulation of metabolites (Aziz *et al.*, 2015; Mohd Din *et al.*, 2021), suggesting that a fixed metabolic network is incapable of maintaining a stable state, even in complex systems. Among the 13 core OTUs, the abundance of OTUs_9947, 2765, and 2727 was mostly maintained and they kept their dominant states (Fig. 4A). They belonged to *Acinetobacter* and *Comamonas* (Supplementary Fig. S3), and specific strains belonging to these genera are known as phenol-utilizing bacteria with higher growth activity (Futamata *et al.*, 2001a, 2005; Aziz *et al.*, 2015). Therefore, these bacteria appear to predominantly utilize phenol, whereas the simulation of the rank-abundance distribution showed that all bacteria corresponding to high ranks did not necessarily exhibit a strong preference for phenol (Fig. 3). These results are consistent with our previous culture-dependent findings (Aziz *et al.*, 2021); a bacterium becomes dominant by microbial cheating of public goods (Smith and Schuster, 2019) supplied from minor populations, indicating that all dominant OTUs did not necessarily utilize phenol. OTU_4642 and 7749 belonged to *Acinetobacter* and *Pseudomonas*, and specific strains belonging to these genera are known as phenol utilizers (Watanabe *et al.*, 1996; Futamata *et al.*, 2001a, 2005; Aziz *et al.*, 2015, Suzuki *et al.*, 2016); however, they remained a minority (Fig. 4B). The rank-abundance distribution suggested that minorities exhibited a weaker preference for phenol and coexisted with majorities, challenging the common assumption that this preference is overcome under competitive conditions. Previous culture-dependent research demonstrated that a minor phenol-utilizing bacterium suppressed phenol hydroxylase, but expressed catechol dioxygenase under coexisting conditions, resulting in the obviation of competitive conditions for phenol, while minor populations incurred the cost for catechol degradation to maintain the functional stability of the whole system (Aziz *et al.*, 2021). A previous study reported that minorities (abundance as low as 0.1%) played roles in the central hub and communication, maintaining the stability and functionality of the microbial community (Guo *et al.*, 2022). These findings suggest that OTU_4642 and 7749 incurred the cost for catechol degradation and supplied common goods, contributing to the maintenance of stable states. The dynamics of middle-class OTUs differed from each other (Fig. 4A and B), which may change not only the metabolism of some bacteria in communities, but also interspecies interactions. The dynamics of middle-class OTUs may release the fixed metabolism of the whole system and contribute to the maintenance of stable states because all core OTUs exhibited specific resilience force (Fig. 4F). These results indicate that core OTUs, which had coexisted and occupied more than 98% of total abundance, functioned for the robustness of the bacterial community under the stable state.

In contrast, extreme minorities consistently underwent exchanges in every transferred culture (Fig. 5A, B, and C, and Supplementary Fig. S5). Growth activity was not necessarily a factor for the exchange of extreme minorities because disappeared OTUs exhibited similar levels to remaining OTUs (Fig. 5D). Therefore, their survival may be restricted by the metabolites produced from highly abundant OTUs in dynamic metabolic networks. These results suggest that extreme minorities play a role as final scavengers for changeable metabolites, contributing to the plasticity of the bacterial community in the stable state. Fluctuations in the three quantitative parameters appeared to accurately reflect community physiological conditions.

In observations of the changing symptoms of stable states, we focused on community vulnerability and resilience forces. A weak force was evident during the 7^th^ to 9^th^ transferred cultures, coinciding with changes in the abundance of middle-class OTUs (Fig. 4B) and the strong exchange of extreme minorities (Supplementary Fig. S4C). The average growth activity of total OTUs was <1.0 in the 10^th^ transferred culture, similar to the level observed during the succession state (Table 1). The community structure underwent tentative changes from the 10^th^ to 12^th^ cultures (Fig. 1A). Community-changing and -forming activities in the 10^th^ transferred culture were not as high as those in the succession state, and resilience force was re-exhibited, suggesting dynamic metabolic network functions. A more detailed understanding of the permissible range of fluctuations for stabilizing microbial ecosystems is crucial, and the driving force behind bacterial community succession, which changes from one stable state to another, remains unknown. Quantitative ana­lyses are expected to provide valuable information for solving this problem using diverse complex microbial samples. These aspects are currently under investigation in our laboratory.

## Citation

Honjo, M., Suzuki, K., Katai, J., Tashiro, Y., Aoyagi, T., Hori, T., et al. (2024) Stable States of a Microbial Community Are Formed by Dynamic Metabolic Networks with Members Functioning to Achieve Both Robustness and Plasticity. *Microbes Environ ***39**: ME23091.

https://doi.org/10.1264/jsme2.ME23091

## Supplementary Material

Supplementary Material

## Figures and Tables

**Table 1.** Quantitative parameters calculated with growth activities of common OTUs

From “t” to “t+1” cultures	Number of OTUs	Number of common OTUs*^a^*	Percentage (%)*^b^*	Average growth activities of all OTUs*^c^*	Community-changing activity*^d^*	Community-forming activity*^e^*
Soil	483	477±3.5	100	5.9±0.15	160±20	40,000±1,340
1^st^	541±6.2		96.5±0.20			
1^st^	541±6.2	437±17	99.3±0.155	0.35±0.73	180±18	180±20
2^nd^	453±15		99.7±0.101			
2^nd^	453±15	156±13	97.2±0.282	0.34±0.79	27±2.9	175±11
3^rd^	179±12		99.4±0.152			
5^th^	65±19	42±8.2	99.8±0.0767	1.0±0.041	3.8±0.98	110±2.6
6^th^	54±6.7		99.9±0.0365			
6^th^	54±6.7	38±3.1	99.9±0.0828	1.2±0.20	3.4±0.80	104±1.6
7^th^	46±2.6		99.9±0.0714			
7^th^	46±2.6	37±2.0	99.9±0.058	4.5±1.7	10±0.90	250±88
8^th^	57±3.2		99.3±0.341			
8^th^	57±3.2	42±7.5	99.5±0.683	3.1±1.4	8.0±3.1	220±50
9^th^	57±10		99.6±0.241			
9^th^	57±19	34±3.8	99.6±0.231	0.70±0.79	6.0±1.2	130±18
10^th^	50±7.0		99.7±0.439			

*^a^*: Number of common OTUs between “t” and “t+1” transferred cultures. *^b^*: Percentage that common OTUs occupy in total abundance. *^c^*: Average of all OTU growth activities between “t” and “t+1” transferred cultures. See the Materials and Methods section. *n*=3. *^d^*: Activities from “t” to “t+1” transferred cultures. See the Materials and Methods section. *n*=3. *^e^*: Activities from “t” to “t+1” transferred cultures. See the Materials and Methods section. *n*=3.
