# Supplementary material for: Stable States of a Microbial Community Are Formed by Dynamic Metabolic Networks with Members Functioning to Achieve Both Robustness and Plasticity
Source: Microbes Environ. 2024 Mar 28;39(1):ME23091. doi: 10.1264/jsme2.ME23091 (PMC10982111; doi:10.1264/jsme2.ME23091)
Supplement: Supplementary file 1 — Supplementary Material [file 39_23091_s1.pdf]

Supplementary Figure S1

(a) Vulnerable force

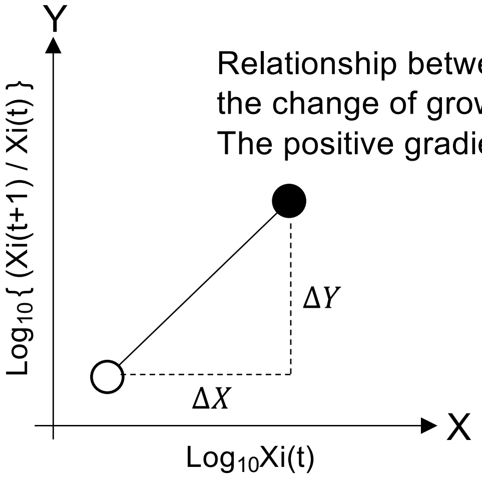

Relationship between the change of abundance (ΔX) and the change of growth activity (ΔY) is positive.
The positive gradient is higher, vulnerable force is stronger.

(b) Resilience force

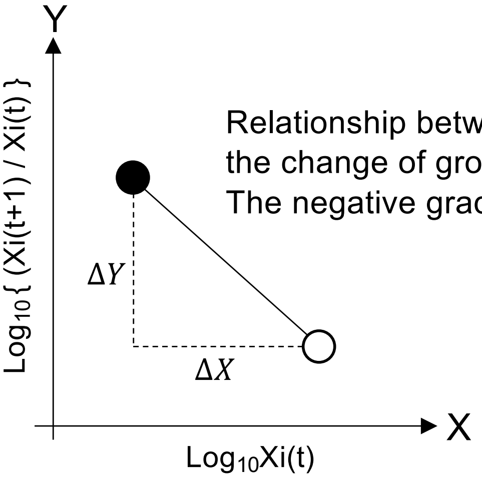

Relationship between the change of abundance (ΔX) and the change of growth activity (ΔY) is negative.
The negative gradient is higher, resilience force is stronger.

Supplementary figure S2

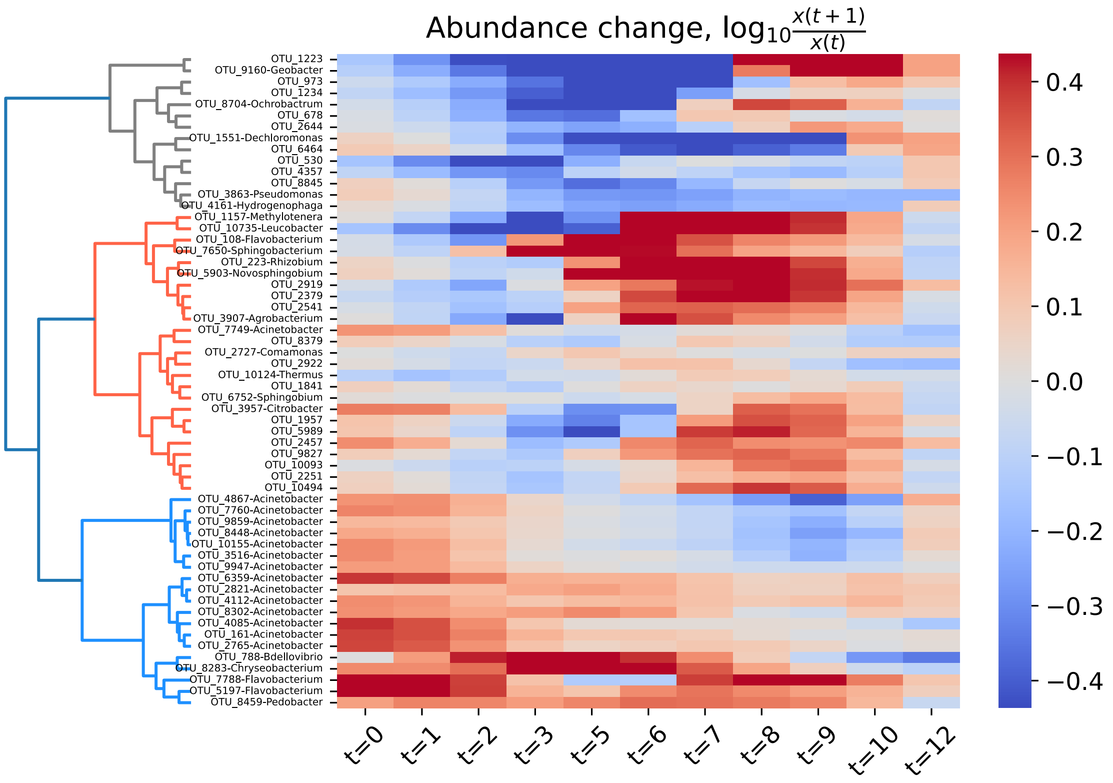

A clustering analysis based on this experimental data indicated that the growth activities of common OTUs detected in one of the three replications at least from the soil sample to the 15th transferred cultures were grouped into 3 clusters: 1) Pronounced growth activity in the early stages, 2) Pronounced growth activity in the later stages, 3) Negative growth activity throughout the experiment, which are shown in blue, red, and gray clusters, respectively. "*t* = 0" indicates soil sample.

Supplementary figure S3

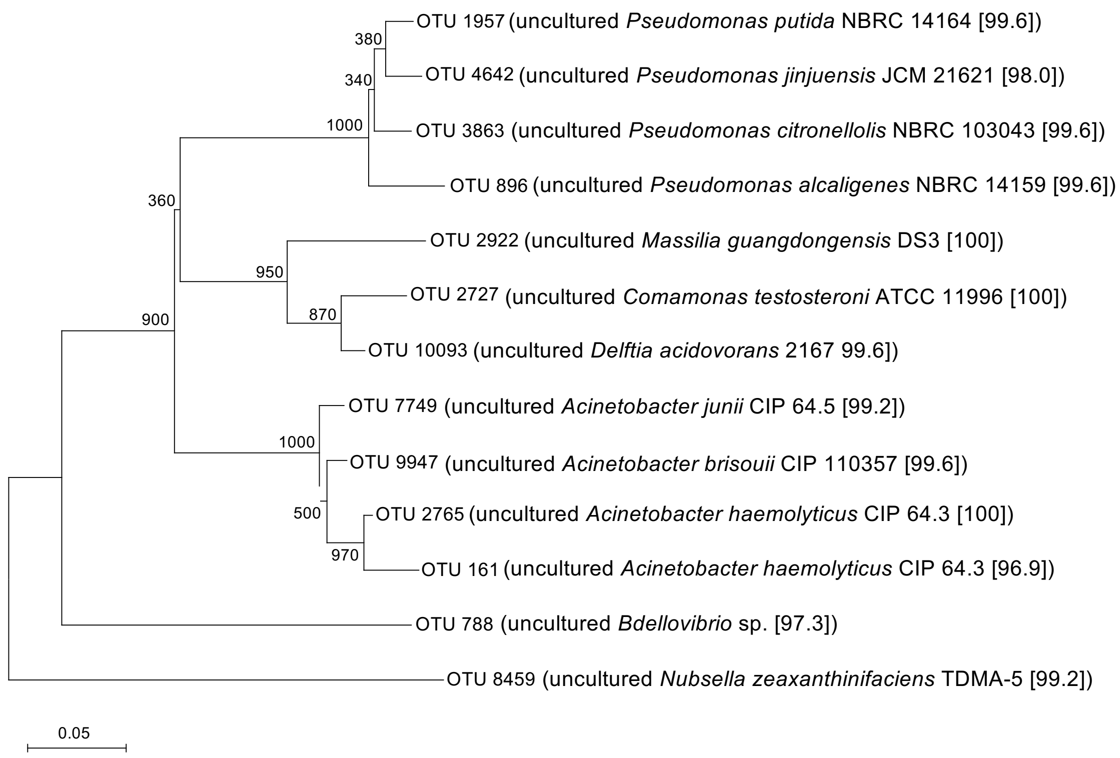

Supplemental Figure S3. Unrooted neighbor-joining tree based on the nucleotide sequences of partial 16S rRNA genes corresponding to the core OTUs.
Most related microorganisms were described in parentheses. The number shown in a bracket indicates percentage of homology. Numbers at the branch nodes are bootstrap values (per 1000 trials). There is no gap in any sequences. The bar represents 0.05 substitution per site.

Supplementary Figure S4

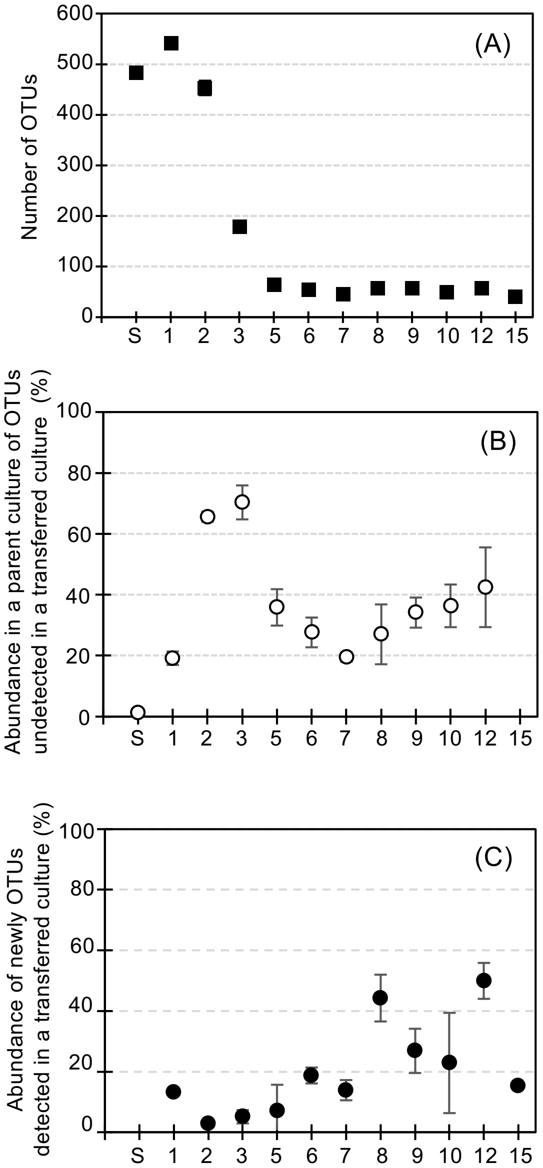

Figure S4. Changes of OTUs abundance.
(A) Number of OTUs in soil and transferred cultures, (B) Abundance in a parent culture of OTUs undetected in the transferred cultures, (C) Abundance of OTUs newly detected in a transferred culture. All symbols mean average of abundance, and error bar means standard deviation (n = 3).

Supplementary Figure S5

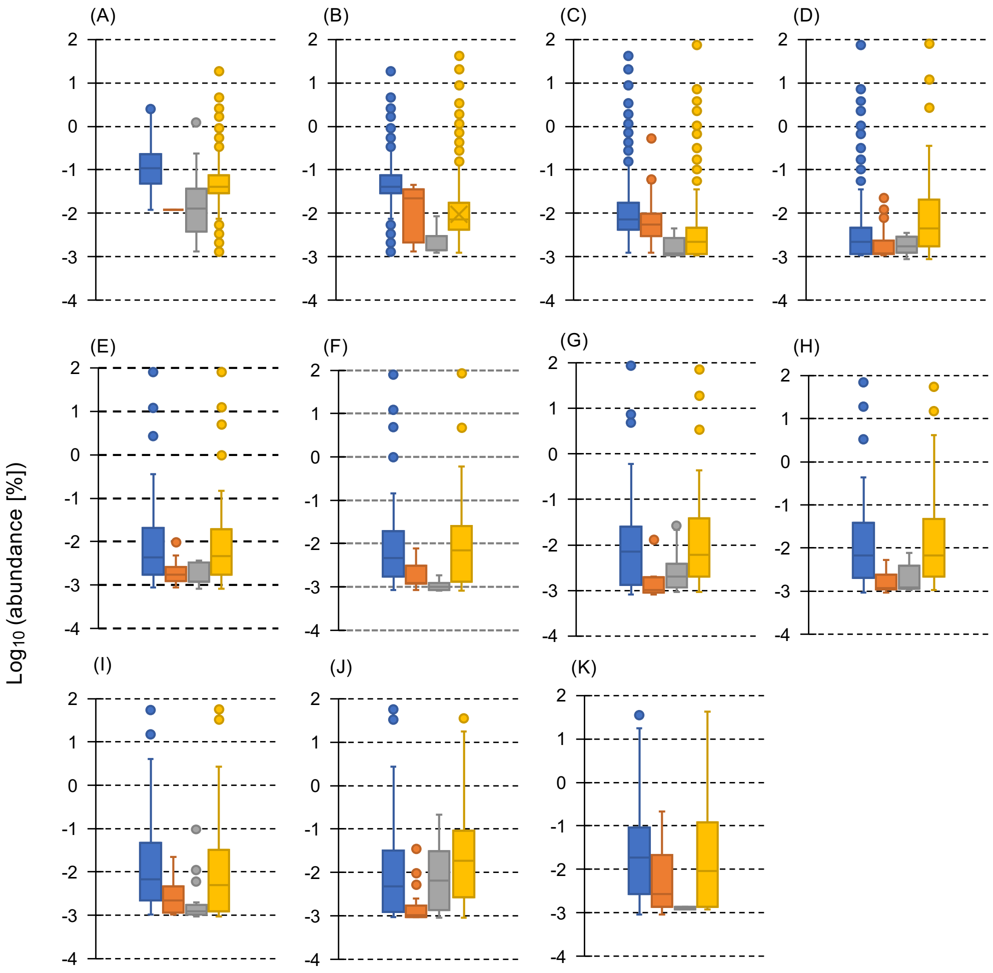

Supplemental Figure S5. Succession of bacterial populations as OTUs level.

Abundance of total OTUs in a parent culture, disappeared OTUs, newly appeared OTUs, and total OTUs detected in a transferred culture.

(A) From soil to the 1st transferred cultures, (B) From the 1st to the 2nd transferred cultures, (C) From the 2nd to the 3rd transferred cultures, (D) From the 3rd to the 5th transferred cultures , (E) From the 5th to the 6th transferred cultures, (F) From the 6th to the 7th transferred cultures, (G) From the 7th to the 8th transferred cultures, (H) From the 8th to the 9th transferred cultures, (I) From the 9th to the 10th transferred cultures , (J) From the 10th to the 12th transferred cultures, (K) From the 12th to the 15th transferred cultures. Blue bars mean abundance of total OTUs in parent cultures, red bars mean abundance in parent cultures of OTUs undetected in the transferred cultures, gray bars mean abundance of OTUs newly detected in the transferred cultures, yellow bars mean abundance of OTUs in the transferred cultures.

Supplementary Table S1. Population dynamics on OTUs level from the 5th to the 10th transferred cultures

#OTU ID	5th	6th	7th	8th	9th	10th
OTU76	–	–	–		–	–
OTU108	–	–	–			
OTU161						
OTU201	–	–		–	–	–
OTU223		–	–			
OTU381	–		–	–	–	–
OTU425	–	–	–	–		
OTU492		–	–	–	–	–
OTU530		–				
OTU678		–	–	–		–
OTU788						
OTU896						
OTU973					–	
OTU1084	–	–	–		–	–
OTU1234		–	–		–	
OTU1242			–	–		–
OTU1313	–	–		–	–	
OTU1330			–	–		–
OTU1338	–	–	–		–	
OTU1450	–		–	–	–	–
OTU1551			–	–	–	–
OTU1578	–	–	–		–	
OTU1599			–		–	–
OTU1671	–	–		–	–	–
OTU1826		–	–	–	–	–
OTU1841						–
OTU1940			–	–	–	–
OTU1957						
OTU2004			–	–	–	–
OTU2043		–	–	–	–	–
OTU2144		–	–			
OTU2251						
OTU2298		–	–		–	–
OTU2379						
OTU2404		–				
OTU2457	–	–	–	–		–
OTU2503		–	–	–	–	
OTU2541						
OTU2572		–	–	–	–	–
OTU2609	–	–	–	–		–
OTU2644			–		–	–
OTU2689			–	–	–	–
OTU2700		–				–
OTU2701		–		–	–	–
OTU2705		–	–			–
OTU2712	–	–	–		–	–
OTU2714	–		–	–		–
OTU2727						
OTU2765						
OTU2821						
OTU2919	–		–	–		
OTU2922						
OTU3035	–		–	–	–	–
OTU3063		–	–	–	–	–
OTU3293						
OTU3390	–	–	–	–		–
OTU3496	–	–	–	–	–	
OTU3516						–
OTU3595						
OTU3863						
OTU3907	–	–	–	–		–
OTU3957				–	–	
OTU3979	–	–	–	–	–	
OTU4042						–
OTU4085						
OTU4112						
OTU4161						
OTU4197	–	–	–	–	–	
OTU4357					–	
OTU4391						
OTU4396	–	–	–	–	–	–

#OTU ID	5th	6th	7th	8th	9th	10th
OTU4401						
OTU4465						
OTU4586						
OTU4642						
OTU4782						
OTU4849	–	–	–			–
OTU4867					–	–
OTU5110						
OTU5169		–	–	–	–	–
OTU5197		–	–			
OTU5237		–	–	–	–	–
OTU5399	–	–	–	–		
OTU5470		–	–	–	–	–
OTU5875			–			–
OTU5876		–	–	–		
OTU5903	–					
OTU5967	–	–	–			
OTU5976	–	–	–			
OTU5989	–	–	–	–		
OTU6038						
OTU6057			–	–	–	–
OTU6074						–
OTU6112	–	–			–	–
OTU6320	–	–	–	–	–	
OTU6359						
OTU6396	–	–	–		–	–
OTU6464		–	–		–	–
OTU6737	–	–	–	–	–	
OTU6752		–				–
OTU6825			–	–	–	–
OTU7164			–			
OTU7650	–					
OTU7749						
OTU7760						
OTU7788		–	–	–	–	–
OTU7794		–	–	–	–	–
OTU7806	–	–	–	–		–
OTU7981	–	–	–		–	–
OTU8098						
OTU8283						
OTU8302			–			–
OTU8379						
OTU8448						
OTU8459						
OTU8528		–	–	–	–	–
OTU8531		–	–	–	–	–
OTU8551			–			–
OTU8704				–		
OTU8726	–	–	–	–	–	
OTU8845		–		–	–	
OTU9026						
OTU9107	–	–	–			–
OTU9276						
OTU9334	–	–	–		–	–
OTU9481	–	–		–		
OTU9609						–
OTU9827	–		–			
OTU9848	–		–	–	–	–
OTU9859						
OTU9947						
OTU10057		–		–	–	–
OTU10093						
OTU10124						
OTU10155						
OTU10263	–		–	–	–	–
OTU10481		–	–	–		–
OTU10494			–			
OTU10594			–		–	–
OTU10735	–	–	–	–	–	
OTU10821		–	–	–	–	–

The abundance indicates average of abundance in triplicated samples. Black; abundance ≥ 1%, gray; 0.1% ≤ abundance < 1%, blue; 0.01% ≤ abundance < 0.1%, green; 0.001% ≤ abundance < 0.01% and white; abundance < 0.001%. These values are average of abundance in triplicated cultures including undetected samples. "–"; Not detected in all triplicated samples. The OTU colored with yellow means the core OTU that was detected in all triplicated samples from the 5th to the 10th transferred cultures.

Supplementary material 1

Parameters used for the simulation

In the simulation of the consumer-resource model with metabolic leakage, we assumed that there two types of resources (phenol and its metabolites) and 200 species. It was assumed that these 200 species are categorized into three distinct groups, with species in the same group having similar metabolic preference, c_{ia}. The numbers of species in the three groups are 50, 50, and 100, respectively.

We set D_{ba} to be $D_{ba} = \begin{pmatrix} 0.5 & 0.0 \\ 0.5 & 1.0 \end{pmatrix}$, where $D_{10} = 0$ because we assumed that phenol is not converted to the secondary product within bacteria. For c_{ia}, we first constructed a group-level preference matrix,

$$c_{Ia} = \begin{pmatrix} 1.8 & 0.2 \\ 0.3 & 1.5 \\ 0.8 & 0.8 \end{pmatrix},$$

where the three rows correspond to the three groups of species. Then, for a species i belonging to the I-th group, c_{ia} is determined by multiplying to c_{Ia} a random normal variable with mean 1 and variance 0.1. For the other parameters, we set $l_a = 1$, $w_a = 1$, $m_i = 0$, and g_i for each i is drawn from a normal distribution with mean 1 and variance 0.17.
